# On the construction of a large-scale database of AI-assisted annotating lung ventilation-perfusion scintigraphy for pulmonary embolism (VQ4PEDB)

**DOI:** 10.3389/fnume.2025.1632112

**Published:** 2025-07-17

**Authors:** Amir Jabbarpour, Eric Moulton, Sanaz Kaviani, Siraj Ghassel, Wanzhen Zeng, Ramin Akbarian, Anne Couture, Aubert Roy, Richard Liu, Yousif A. Lucinian, Nuha Hejji, Sukainah AlSulaiman, Farnaz Shirazi, Eugene Leung, Sierra Bonsall, Samir Arfin, Bruce G. Gray, Ran Klein

**Affiliations:** ^1^ Department of Physics, Carleton University, Ottawa, ON, Canada; ^2^ Electrical Engineering and Computer Science, University of Ottawa, Ottawa, ON, Canada; ^3^ Research & Development, Jubilant DraxImage, Kirkland, QC, Canada; ^4^ Division of Nuclear Medicine and Molecular Imaging, Faculty of Medicine, University of Ottawa, Ottawa, ON, Canada; ^5^ Department of Nuclear Medicine and Molecular Imaging, The Ottawa Hospital, Ottawa, ON, Canada; ^6^ Department of Radiology and Nuclear Medicine, Hôpital Maisonneuve-Rosemont, Montréal, QC, Canada; ^7^ McGill University Faculty of Medicine, Rue de la Montagne, Montréal, QC, Canada; ^8^ Department of Nuclear Medicine, Jewish General Hospital, Montreal, QC, Canada; ^9^ Department of Medical Imaging, St. Michael’s Hospital, Unity Health Toronto, Toronto, ON, Canada

**Keywords:** database, image annotation, crowdsourcing, ventilation-perfusion scintigraphy, pulmonary embolism

## Abstract

**Introduction:**

Ventilation-perfusion (V/Q) nuclear scintigraphy remains a vital diagnostic tool for assessing pulmonary embolism (PE) and other lung conditions. Interpretation of these images requires specific expertise which may benefit from recent advances in artificial intelligence (AI) to improve diagnostic accuracy and confidence in reporting. Our study aims to develop a multi-center dataset combining imaging and clinical reports to aid in creating AI models for PE diagnosis.

**Methods:**

We established a comprehensive imaging registry encompassing patient-level V/Q image data along with relevant clinical reports, CTPA images, DVT ultrasound impressions, D-dimer lab tests, and thrombosis unit records. Data extraction was performed at two hospitals in Canada and at multiple sites in the United States, followed by a rigorous de-identification process. We utilized the V7 Darwin platform for crowdsourced annotation of V/Q images including segmentation of V/Q mismatched vascular defects. The annotated data was then ingested into Deep Lake, a SQL-based database, for AI model training. Quality assurance involved manual inspections and algorithmic validation.

**Results:**

A query of The Ottawa Hospital's data warehouse followed by initial data screening yielded 2,137 V/Q studies with 2,238 successfully retrieved as DICOM studies. Additional contributions included 600 studies from University Health Toronto, and 385 studies by private company Segmed Inc. resulting in a total of 3,122 V/Q planar and SPECT images. The majority of studies were acquired using Siemens, Philips, and GE scanners, adhering to standardized local imaging protocols. After annotating 1,500 studies from The Ottawa Hospital, the analysis identified 138 high-probability, 168 intermediate-probability, 266 low-probability, 244 very low-probability, and 669 normal, and 15 normal perfusion with reversed mismatched ventilation defect studies. In 1,500 patients were 3,511 segmented vascular perfusion defects.

**Conclusion:**

The VQ4PEDB comprised 8 unique ventilation agents and 11 unique scanners. The VQ4PEDB database is unique in its depth and breadth in the domain of V/Q nuclear scintigraphy for PE, comprising clinical reports, imaging studies, and annotations. We share our experience in addressing challenges associated with data retrieval, de-identification, and annotation. VQ4PEDB will be a valuable resource to development and validate AI models for diagnosing PE and other pulmonary diseases.

## Introduction

The incidence of pulmonary embolism (PE) spans from 39–115 per 100,000 annually (1). Ventilation-Perfusion (V/Q) nuclear scintigraphy has long been a modality of choice for evaluating patients with suspected PE and post treatment follow up (2, 3). V/Q scans are also a promising modality in diagnosing other pathologies such as CTEPH (4) and COPD (5). Over the past two decades the community has transitioned from planar scintigraphy to 3D single photon emission computed tomography (SPECT) due to higher sensitivity in segmental defects, while some persist with planar imaging (6, 7). Unlike other medical imaging modalities, automatic PE diagnosis using V/Q scintigraphy had once garnered significant attention for AI applications in the 90s and early 2000s showing excellent results, only to be subsequently abandoned. Therefore, automatic diagnosis of PE using V/Q scans can greatly benefit from modern advancements in deep learning algorithms (8).

The primary objective of this work was to build a multi-center, comprehensive database of imaging and ancillary data of patients with suspected PE and make it known to the community. Our second goal was to share our experience in building the database and crowdsourcing annotations of the data, including challenges encountered in data retrieval from picture archiving and communication system (PACS), imaging data deidentification, clinical report deidentification, data annotation, report homogenizing, and hosting the aggregated data. We share our challenges, solutions, and considerations. We hope that the registry will facilitate data management for research projects to enable collaboration amongst researchers.

## Methods

### Study objective and design

**Figure 1:**
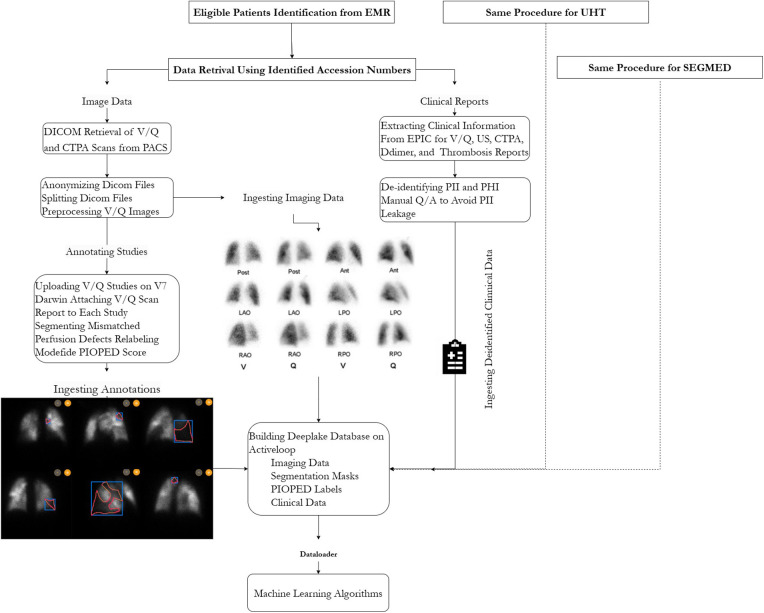
Schematic representation of processing steps for building the VQ4PEDB registry. Note, EMR, electronic medical record.

The creation of the VQ4PEDB database was approved by the research ethics boards (REB) of participating hospitals. The main study is managed under the Clinical Trials Ontario (Project ID 3945). The study objective was to build an imaging registry of patient-level V/Q structured dataset in the form of rich clinical and imaging data, including nuclear medicine physician narrative and impression, CTPA clinical report and imaging files, pre-scan ultrasound for deep vein thrombosis (DVT) impression, D-dimer lab test, thrombosis unit report, and annotations of V/Q images. The workflow of this study is illustrated in Figure 1.

### Internal data extraction

In consultation with The Ottawa Hospital (TOH) Data Warehouse, we defined a query of the electronic medical record (EMR) database with the following inclusion criteria:

• All patients referred to the nuclear medicine department for a lung V/Q study between June 1, 2019 and February 28, 2023
• All CTPA studies within ±1 year of V/Q scan
• All Chest CT studies within ±1 year of V/Q scan
• All ultrasounds for DVT within −7 to +21 days of V/Q scan
• All D-dimer within ±3 days of V/Q scan
• All thrombosis reports within 14 days post V/Q scan

Results were returned in an Excel file (Supplementary Material I) including corresponding sheets for each data type in tidy data table format. This Excel file sample included patient demographics, medical record number (MRN), accession number, ordering department, order date and time, procedure date and time, exam reason, narrative, findings, and impression columns for different V/Q, CTPA, US, D-dimer, and thrombosis sheets.

To extract imaging data, we developed a bash script to automatically retrieve V/Q and CTPA DICOM studies tailored with each MRN and accession number from our PACS. Data transfer to a local computer was achieved using the DICOM query/retrieve network protocol. Thus, the PACS team only needed to aid in establishing a DICOM node, and monitoring traffic during testing to ensure that clinical service would not be disrupted.

### External data extraction

Patient data from University Health Toronto (UHT) was extracted for the period between August 12, 2016, and February 9, 2024. This dataset included ventilation/perfusion (V/Q) scans, computed tomography pulmonary angiography (CTPA) scans, and associated clinical reports. Inclusion criteria for UHT data required patients to have undergone both V/Q and CTPA imaging within a clinically relevant timeframe, along with available clinical documentation supporting evaluation for suspected PE. The third dataset for high probability and intermediate probability scans [as determined by PIOPED criteria (9)] was purchased from Segmed Inc., a private data collector company, which collected these data from 17 unique centers between 2010 and 2023.

### Anonymization of DICOM files and deidentification of clinical reports

For The Ottawa Hospital, all imaging data were stored in DICOM format and automatically anonymized using a custom in-house script. This script used a multi-step approach to remove patient PII/PHI DICOM tags, as detailed in Supplementary Material II and then removed all private DICOM tags as defined by the DICOM NEMA standard. As a further measure, we visually inspected sample deidentified data from at least one case for each camera type to ensure no remaining private tags and no PII/PHI in the remaining fields. Additionally, each patient's Medical Record Number (MRN) was replaced with a unique study ID generated by the data warehouse. Study ID and accession numbers were used to link patient data from the different sources. Furthermore, all secondary screen captures were excluded from the database as they pose a risk of containing patient identifiers in the binary image data. Subsequently, planar scintigraphic projection DICOM files, which commonly contain acquisition of two projections (e.g., anterior and posterior) were split to have one DICOM file per projection using a custom Python script. This was done to comply with the annotation platform's requirement of one unique DICOM file and DICOM Series UID per image visualization slot. Since each patient might have multiple imaging studies, DICOM files were organized into separate folders based on the study ID and accession number.

For clinical report texts, we adopted and compounded the effect of the following three independent approaches as a conservative de-identification strategy: (1) Segmed Inc.'s Python-based web server was used to remove PII/PHI from clinical reports, (2) RegEx rules were used to remove Canadian formatted addresses and postal codes in Python, and (3) resulting texts were fed to a Microsoft Copilot agent that was instructed to list suspected people names, addresses, street names, 5–8 digit numbers, business names, clinic names and occupations. The agent was further prompted to ignore medical terms. The resulting terms were then manually screened for relevance, the terms were searched for in the text and then replaced with “[Anon]”.

At UHT, data extraction was performed using Health Canada-approved software for anonymization of DICOM files and deidentification of clinical text reports was conducted manually by onsite technologists before transfer of the data. As for dataset provided by Segmed Inc., Segmed Inc. is already HIPAA compliant, and it is assumed that patient privacy measures have been adequately addressed. Nevertheless, as an extra layer of security against PII/PHI leaks, DICOM files and clinical text reports from UHT and Segmed Inc. were also processed using the in house-built Python scripts at the core lab (TOH).

### Annotating V/Q images

**Figure 2:**
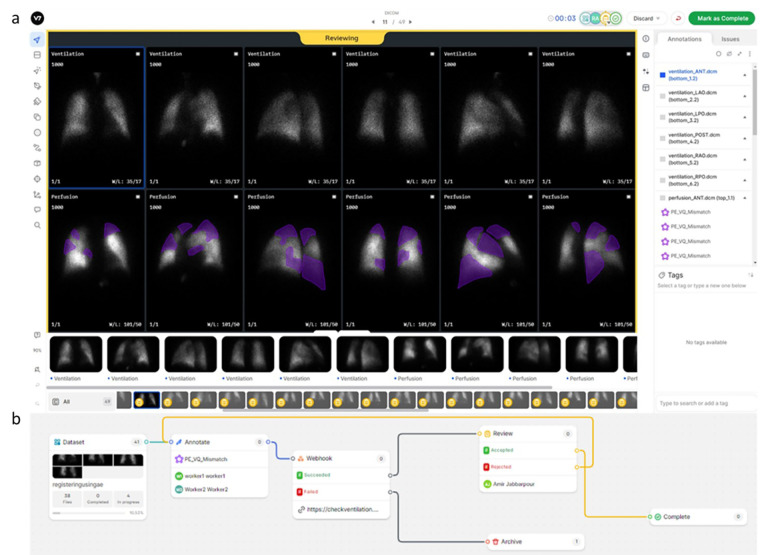
**(a)** Example of annotation using the V7 viewer, displaying paired V/Q planar images in six projections with basic image display adjustments and annotation tools. Communication features for discussing ambiguous cases are also illustrated. **(b)** Illustration of the image dataset annotation workflow configuration on the V7 platform including splitting ratios for annotators, webhooks for custom sanity checks or cloud storage of annotations, and consensus among annotators.

To annotate this large number of studies, we resorted to a distributed crowd sourcing approach using the V7 Darwin internet hosted platform (V7 Labs, London, UK). The V7 viewer was configured to display paired V/Q planar images in 6 projections (anterior, posterior, and 4 oblique views), emulating our clinical viewer (Figure 2a). The V7 viewer has basic functionality for controlling image display (intensity saturation, contrast, brightness, window length, window width, zoom, and colormap). In addition, annotation tools are available including segmentation and labeling. Segmentation overlay display can be controlled including display toggling and opacity. Lastly, V7 offers commenting tools to communicate with study leads, for example, regarding ambiguous cases or tasks.

The V7 platform also offers versatile workflow tailoring with custom steps, e.g., consensus among annotators, splitting ratios for annotators, webhook for either custom sanity checks or storing annotations in cloud to manage study design (Figure 2b). AI models also can be integrated into V7 workflow to automatically generate segmentations which later could be modified by annotators. V7 also provides statistical metrics for performance of each annotator on dataset and individual basis that are useful for tracking individual annotator performance and financially compensating them. De-identified data were uploaded to a Microsoft Azure blob storage and then pushed to V7 cloud.

Annotators were tasked with segmenting vascular V/Q mismatch defects on all relevant projections. Furthermore, annotators recorded their perceived risk of PE using the modified PIOPED criteria (Table 1) using the imaging data and accompanying interpreting physician report from the clinic (10). Baseline and follow-up V/Q examinations in this study were originally classified as chronic PE but were treated as acute PE during the annotation and relabeling process. According to modified PIOPED, annotators also labeled studies into one of the following categories with regards to likelihood of PE: high, intermediate, low, very low, normal, or normal perfusion with ventilation reverse mismatches (11). Annotations were performed in several stages with different batch sizes to train, qualify and audit each annotator, and to fine tune the instructions to the annotators with the aim of minimizing task ambiguity.

**Table 1:** Summarized modified PIOPED category used for re-scoring studies. Note, CXR is assumed to be normal for all patients.

Category	Conditions (any condition qualifies)
High	• Two large segmental perfusion defects without ventilation or chest x-ray abnormality • One large and two moderate perfusion defects • Four moderate perfusion defects
Intermediate	• One moderate or less than two large defects • Corresponding lower lung zone defect and chest x-ray abnormality • Ventilation-perfusion defects and small effusion • Difficult to categorize as high or low probability
Low	• Defect with larger chest x-ray abnormality • Fewer than three small segmental defects
Very low	• Nonsegmental perfusion defect • Perfusion defect smaller than chest x-ray finding • Stripe sign • Triple match mid/upper lung • Multiple matched defects
Normal	• No perfusion abnormalities
Normal Q with reversed mismatched V defects	• No perfusion abnormalities with reversed significant mismatched ventilation defects

**Figure 3:**
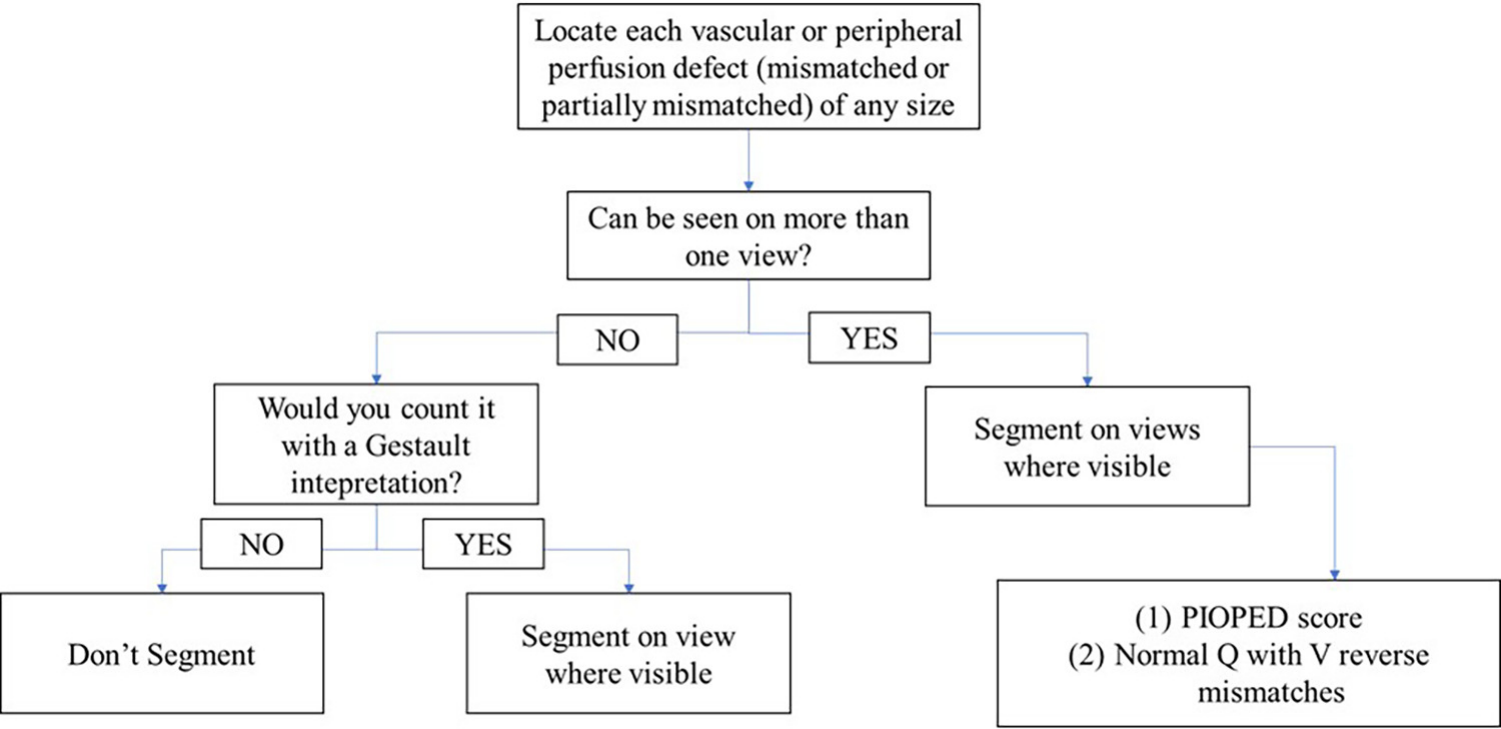
Annotation policy workflow developed for annotators, outlining step-by-step procedures to ensure consistency and accuracy. It includes guidelines for annotation criteria and protocols for handling ambiguous cases.

First, each annotator was given an introduction dataset of 49 patients with high probability PE prevalence, identified based on original impression of the reporting nuclear medicine physician, and the annotator was tasked with segmenting all perfusion defects. Annotations from this dataset were reviewed and analysed with senior nuclear medicine physician along with feedback collected from the annotators. All annotations were visually reviewed by the study lead (AJ and in consultation with staff physicians). Studies with obvious annotations errors—including those with missed defects or false positives—were rejected and returned to the annotators with detailed feedback. This process was facilitated by a built-in feature in V7 Darwin that enables precise communication of findings at any point within any projection, supporting iterative training and improving annotation quality. Based on the annotation results and survey from annotators on this first dataset, tasks were more clearly defined, tutorial materials were produced to guide labeling of ambiguous cases, and the viewer functionality was enhanced. Ambiguous cases were reviewed and discussed with two senior nuclear medicine physicians until a consensus was reached. Improved instructions are listed in Figure 3. Annotators were not remunerated for annotating these data but were financially compensated for all subsequent annotations.

A similar, second dataset (i.e., 49 patients and high PE probability) was created for validating annotators performance. Performance of annotators again were reviewed and analysed. Low-quality annotations were sent back to the original annotator to correct. Annotators whose performance was deemed unacceptable were excluded from the rest of the project.

The third and fourth datasets were uploaded to V7, comprising 500 randomly selected studies. These were assigned to qualified annotators on a first-come, first-to-annotate basis. All annotations were reviewed by the study lead and—where disputed—were sent back to the annotator for further review and refining until a consensus was reached. These data are used to train and validate a preliminary AI for semantic segmentation of vascular perfusion defects.

As a means to accelerate annotation, we developed a deep learning model based on annotated images from the first to fourth datasets to automatically propose candidate vascular perfusion defects on V/Q planar imaging and deployed it on V7. Subsequent studies from TOH and other hospitals will be annotated using this AI model to initialize the segmentations. Annotators are tasked with either approving or correcting the AI-generated candidate segmentations. This approach enables us to iteratively improve the AI with additional data with the aim of eventually developing an AI that can be applied to autonomous image segmentation.

### Data ingestion pipeline

All training data and labels were ingested into Deep Lake on Activeloop (Mountain View, USA), a SQL-based database designed to organize complex unstructured data. Processed imaging data, their metadata, modified PIOPED scores, polygons of perfusion defects, and bounding boxes generated from polygons were ingested into one database, while de-identified clinical reports were ingested separately into different datasets. To utilize the ingested data, an Activeloop-provided API was used to perform single or joined queries to load the required data into the Python environment to train AI models using data loaders.

### Quality control

Imaging and ancillary data were quality checked in several stages of this study to verify the integrity of the study. After de-identification, 10% of DICOM files were randomly sampled and manually inspected to check patients' PII and PHI leakage and update the de-identification algorithm accordingly. Clinical reports were also sampled by 10% and hand-checked to verify the performance of the de-identification algorithm and update it accordingly. Randomized screening of uploaded studies to V7 was performed to ensure having ventilation and perfusion from the same date for patients who had more than one study. Although data ingestion to Deep Lake dataset has been conditioned on study IDs and should prevent confusion of data between patients, Deep Lake dataset was investigated logically for any mismatch between clinical and imaging data in any of study IDs, gender, and patient age. Finally, a script was developed to cross check Deep Lake dataset information with clinical reports and imaging dataset. Any discrepancies were investigated and followed to resolve the issue.

## Results to date

### Extracted studies

**Figure 4:**
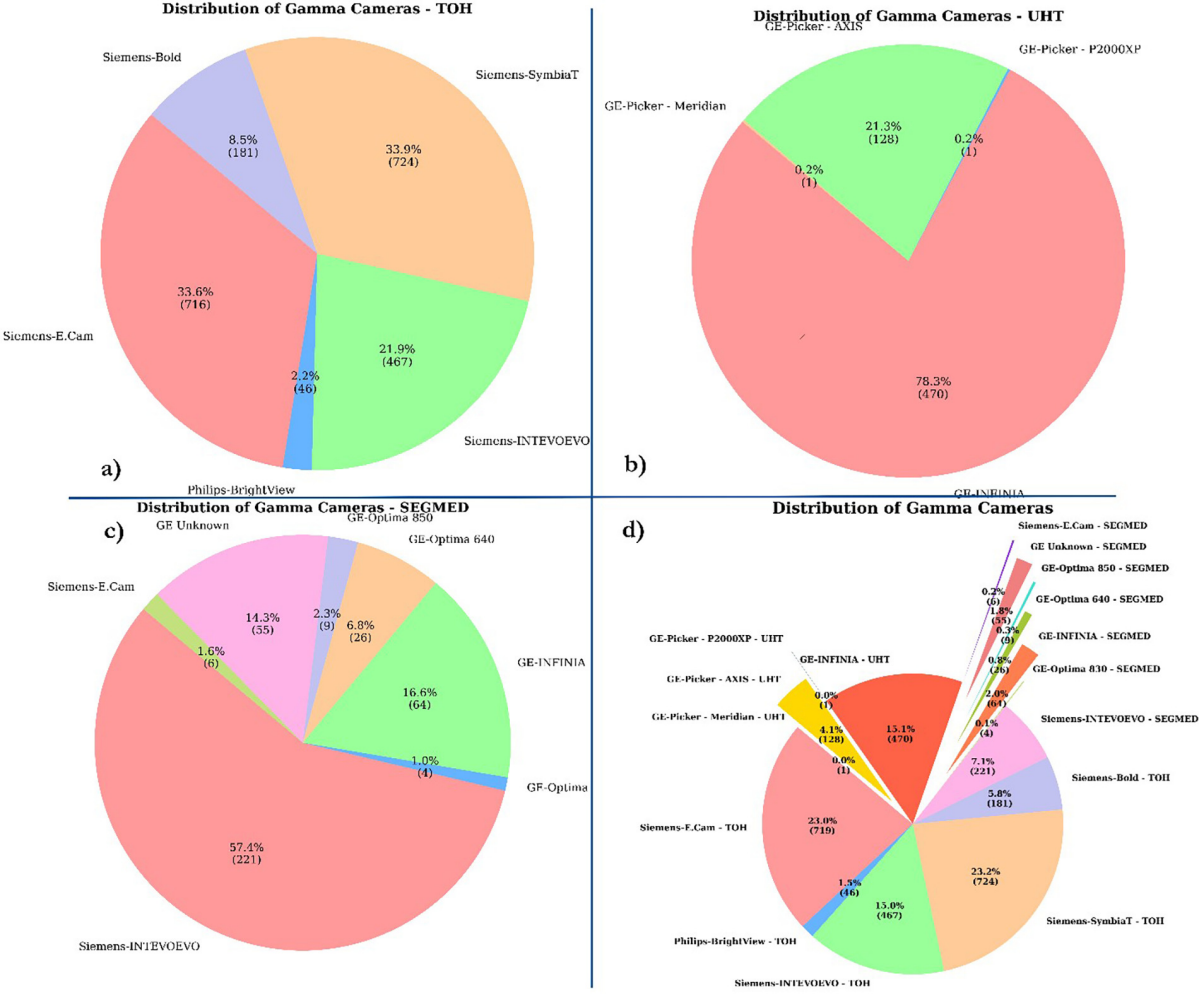
Distribution of camera types. This pie chart illustrates the distribution of different camera types collected from **(a)** TOH **(b)** UHT **(c)** SEGMED **(d)** all centers. The chart shows the percentage and absolute number of each camera type, highlighting their respective contributions to the total.

The data warehouse query from TOH resulted in 2,289 studies and DICOM downloading script successfully retrieved 2,288 studies. Eight studies were excluded from the analysis because they were acquired using an older Mediso Orbiter gamma camera with a circular field of view. Additionally, 152 patients were excluded due to either missing ventilation or perfusion data, or because the images were pseudo-planar. Finally, 2,137 images were included. UHT hospital contributed 600 V/Q planar and SPECT studies. The Segmed Inc. database contributed 385 V/Q images of high and intermediate PIOPED score with corresponding clinical reports collected from 17 different sites. In total, the dataset consisted of 3,119 V/Q planar and SPECT images, many with corresponding clinical reports, CTPA, US, and D-dimer test. The breakdown of number of the included imaging modalities and corresponding reports is presented in Table 2. Additionally, the distribution of included scanners is depicted in Figure 4.

**Table 2:** Summary of number of datasets from each data source.

Datasets\Source	TOH	UHT	Segmed Inc.
SPECT V	N/A	368	N/A
SPECT Q	563	368	N/A
Planar V	2,137	600	371
Planar Q	2,137	600	385
V/Q report	2,137	600	385
CTPA	1,644	N/A	N/A
CTPA report	1,644	39	N/A
US for DVT report	706	115	N/A
D-dimer	501	N/A	N/A
Thrombosis report	2,137	N/A	N/A

### Image acquisition protocols

The included scanner models and the percentage of included studies from each scanner are listed in Table 2. Most of the included studies were acquired using Siemens scanners, including Ecam, Intevo Evo, Symbia, Bold, and the rest were imaged by Philips Brightview. All images were acquired using a dual head gamma camera. All used low energy, high resolution parallel hole collimators.

TOH image acquisition protocol consists of performing ventilation portion in supine position with 370–555 MBq (10–15 mCi) of Technegas (^99m^Tc-Pertechnetate). SPECT image acquisition parameters are matrix size of 128 × 128, zoom 1.0, energy window 140 ± 7.5% [129–150] and lower scatter [108–129] with 64 number of views per head, time per view 15 s, and acquire-during-motion. Perfusion studies were performed in supine position using either with full dose 148–185 MBq (4–5 mCi) of ^99m^Tc-macroaggregated albumin (MAA) or half dose 74–93 MBq (2–2.5 mCi) for pregnant or pulmonary hypertension patients. To properly appreciate true blood perfusion to the lungs, perfusion count rate was ensured to be at least 4 times the ventilation count rate. The SPECT images, if available, were reconstructed using Hermes Hybrid Recon V1.1a or higher with 4 iterations and 8 subsets of OSEM reconstruction algorithm. Attenuation correction was applied using either with CT if available or using synthetic attenuation maps from the scatter. Collimator correction was performed followed by 3D Gaussian 0.8 cm FWHM post filter correction. The ventilation and perfusion static images were acquired in the 6 projections including anterior, posterior, right anterior oblique, left posterior oblique, left anterior oblique, and right posterior oblique using dual head camera with matrix size of 256 × 256, zoom 1.45, energy window 140 ± 7.5% [129–150] keV. Ventilation planar and perfusion planar acquisition was continued until 150 kcts/image or 300 s and 600 kcts/image or 300 s, respectively.

UHT data had a similar acquisition protocol except that they acquired two extra projections, right lateral and left lateral and used a variety of ventilation agents as summarized in Table 3. The three most frequently reported aerosol in this dataset were Technegas (*n* = 2,280), ^99m^Tc-diethylenetriamine pentaacetate (^99m^Tc-DTPA, *n* = 397) and ^99m^Tc-methyl diphosphonate (^99m^Tc-MDP, *n* = 148), respectively. MAA is the only perfusion agent used throughout all centers.

**Table 3:** Summary of ventilation agents used at each data source.

Radiopharmaceutical	Number of studies
TOH	2,137
Technegas	2,137
UHT	600
MDP	148
DTPA	47
MIBI	35
PYP	15
Pyrophosphate	2
Sodium Pertechnetate	1
Myoview	1
N/A	351
Segmed	385
DTPA	350
Xenon-133	12
PYP	9
N/A	14

### Demographics

At TOH, most participants were female, with 1,461 (64%) females compared to 819 (36%) males. The age of participants ranged from 16–101 years, with an average age of 52.89 and 60.54 years females and males, respectively. At UHT, females also had a higher representation, with 379 (63%) females compared to 221 (37%) males. The age range of participants was similar, from 18–104 years, with an average age of 59.13 years for females and 66.57 years for males. Segmed Inc. contributed 342 female (59%) and 240 (41%) male participants to this study with mean age of 65.62 and 67.94 years for females and males, respectively. Patient age was missing for 33 patients in Segmed dataset.

**Figure 5:**
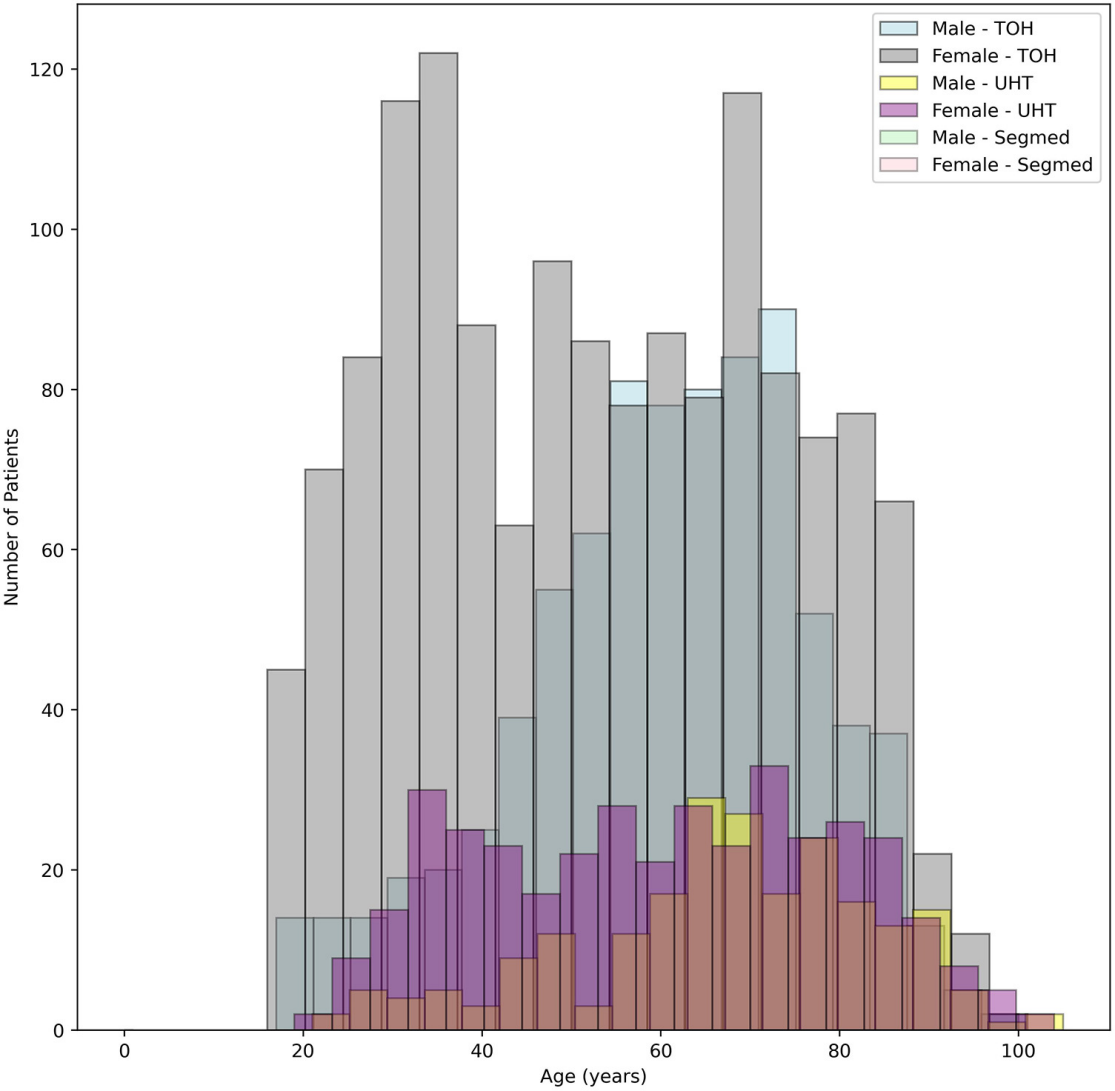
Histogram of age of included patients colour coded for male/female and data source.

**Figure 6:**
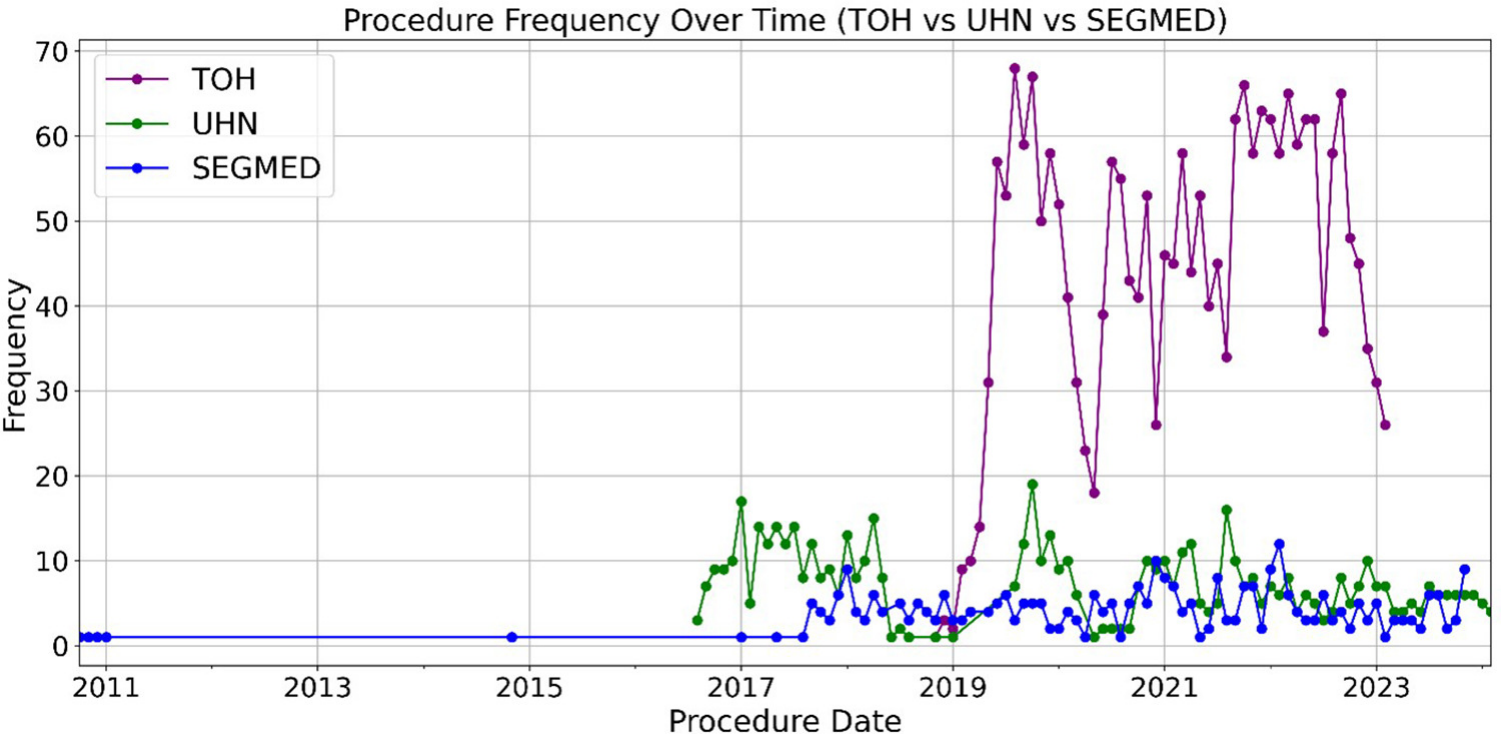
Trends in V/Q acquisition over time. This figure illustrates the temporal evolution of V/Q imaging, demonstrating uniform data collection over the inclusion date range with some variations with a sharp decrease associated with the early phase of the COVID-19 outbreak.

Demographics of patients are represented in Figure 5. The oldest and most recently acquired V/Q studies in this dataset were from Segmed Inc. in 2010 and UHT in 2024, respectively. Finally, Figure 6 illustrates a time distribution of when data were acquired for each of the 3 sources.

### Planar image annotation

**Figure 7:**
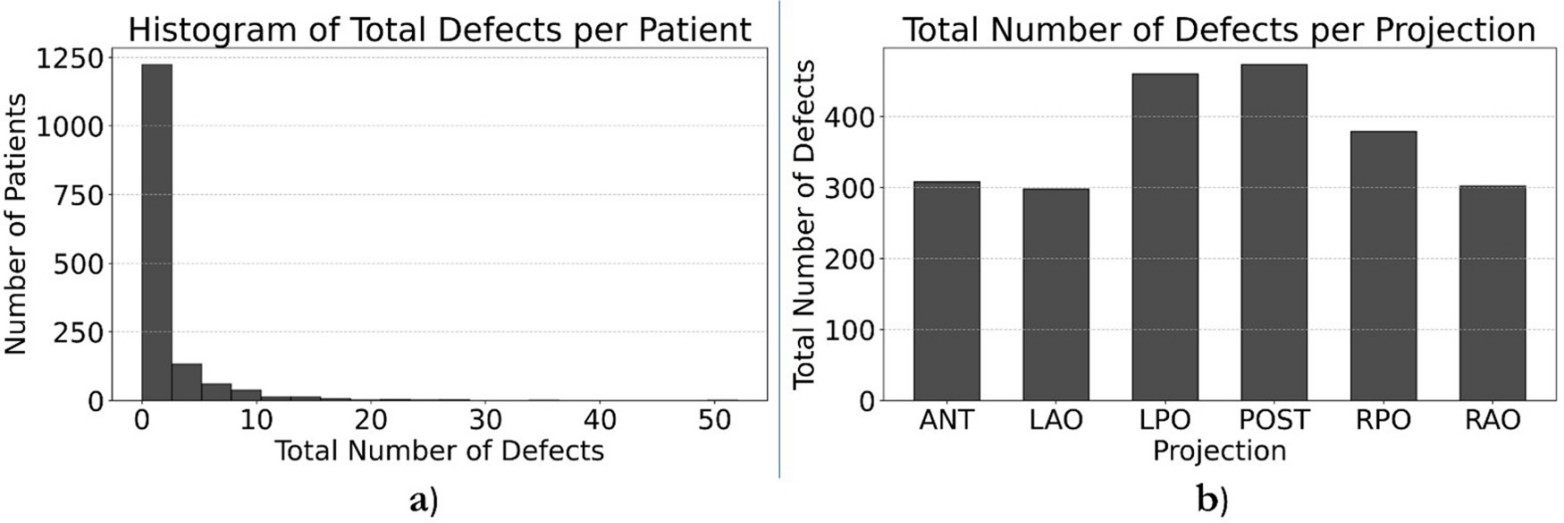
Histogram representation of number of vascular perfusion defects per patient **(a)** and projection **(b)**.

**Figure 8:**
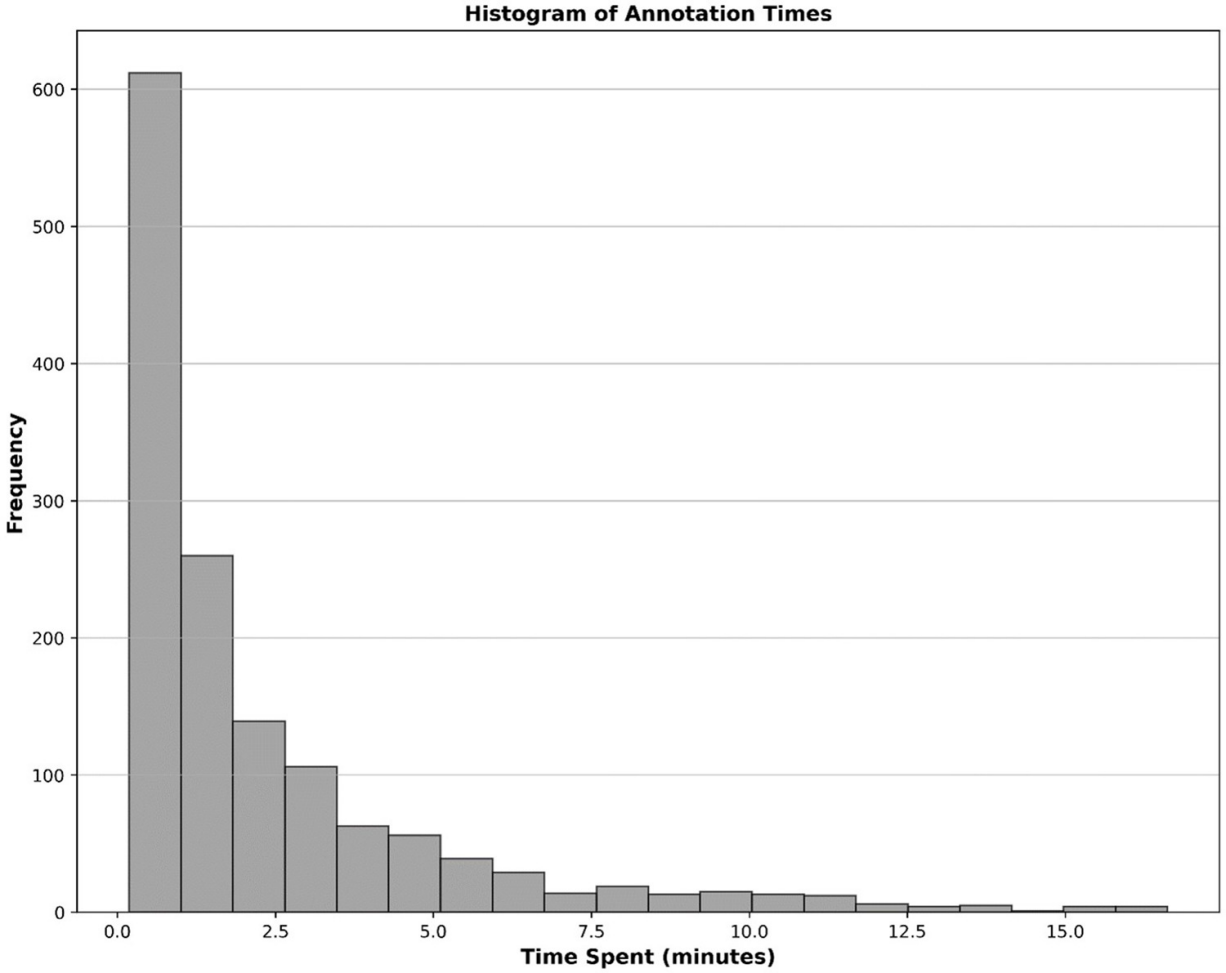
Histogram illustrating the distribution of time spent on annotating cases as automatically recorded by the V7 darwin platform.

We recruited a total of eight nuclear medicine fellows and two senior nuclear medicine physicians for this study. All eight fellows participated in the annotation process. While both senior physicians were involved in reviewing ambiguous cases and providing annotation instructions, one also contributed to labeling process. Five fellows whose annotations evaluated as unsatisfactory or who were unable to continue due to scheduling conflicts were excluded from further participation. Consequently, four annotators were qualified to continue until the fourth dataset, with three fellows and one senior physician for annotating the last dataset. By November 30th, 2024, a total of 1,500 studies had been annotated. Table 4 provides a comprehensive breakdown of the annotation details. Contouring segmental perfusion defects resulted in 3,511 contours in 1,500 studies. Distribution of vascular perfusion defects per patient and projection are depicted in Figures 7a,b, respectively. After removing annotation times more than 1000s as outliers, the average time spent on annotating cases was 3.50 ± 4.20 min, with a total annotation time of 82.53 h across all cases, Figure 8. The time ranged from 0.28 min–24.93 min, with a median of 1.70 min. The interquartile range (IQR) was 0.80–4.49 min.

**Table 4:** Summary of annotations to date from each data source.

Datasets\Source	TOH	UHN	Segmed
Number of studies	1,500	600	385
SPECT V/Q defects	N/A	N/A	N/A
Planar V/Q defects	3,511	N/A	N/A
High	138	72	104
Intermediate	168	12	281
Low	266	200	0
Very low	244	30	0
Normal	669	286	0
Normal Q with reversed mismatched V defects	15	N/A	0

## Discussion

AI powered by machine learning algorithms is dependent on large and diverse dataset examples for training and validations. Data collection and annotation are often the most difficult component of AI development. To that end, we are growing a large imaging and clinical registry dataset of V/Q images including ancillary data crucial to the diagnosis of PE. In doing so, it is our hope to fuel collaborative research that will deliver useful clinical tools to aid in the diagnosis of pulmonary diseases such as PE, COPD, CTEPH, and segmental quantification for pre-operation. There are several publicly available datasets for PE acquired with computed tomography (CT), which have fostered diagnostic AI models for CTPA (12–14). In contrast, the absence of similar datasets for V/Q imaging—due to the associated challenges and difficulties outlined in this work—has hindered progress in V/Q-based AI applications. With the development of VQ4PE-DB, we aim to address this gap and facilitate advancement in the field. In fact, the establishment of VQ4PEDB has already proven its utility. Ghassel et al. investigated the effect of different resizing techniques on similarity metrics, Ghassel et al. developed a deep learning based pseudo-planar V/Q image generation model and Ansari et al. built a bronchopulmonary segment atlas registration strategy on V/Q images as a diagnostic aid for PE, utilizing data directly from the registry (15–18). These projects demonstrated the ability to quick and efficiently extract data meeting specific requirements (e.g., a mixed population of studies with normal PIOPED interpretations, or a balanced set of images in terms of PIOPED criteria. Reasonable data requests can be directed to the senior author. By making this database available to the research community, we hope to accelerate the development of lung V/Q applications.

### PACS data extraction

Like CTPA, V/Q SPECT can detect small subsegmental pulmonary emboli, which, in the absence of clinical complications, may not necessitate therapeutic intervention. V/Q imaging methodology exhibits notable geographic variation: planar imaging continues to be the predominant approach in the United States, whereas V/Q SPECT is endorsed by the European Association of Nuclear Medicine (EANM) and widely adopted across Europe, Australia, and several Asian countries (19). TOH and UHT were identified for this study since, to our knowledge, they are among the limited number of institutions employing a combined V/Q SPECT and planar imaging protocol. To ensure alignment with broader North American standards, Canadian regulatory frameworks often require the inclusion of United States sourced data when evaluating or validating planar V/Q imaging protocols.

The inclusion criteria for each center were tailored according to the scope and practical availability of archived data. TOH provided the most comprehensive dataset, including V/Q scans, CTPA, ultrasound reports, D-dimer results, and thrombosis clinic documentation, due to capabilities for automated data extraction from PACS for imaging data by us (as described in the methods section) and by the data warehouse team for all non-imaging data. In contrast, UHT dataset was limited to V/Q and CTPA records as manual data extraction was required. Although Segmed Inc. offered data across all PIOPED probability categories—including low, very low, and normal—to address class imbalance, we restricted inclusion to high probability and intermediate probability studies, thereby excluding cases with a few or no detectable defects.

At our institution, image retrieval times from PACS could take several minutes per study as older data is backed up to lower tier, long term storage requiring retrieval. For patients whose data was stored in tier 2 storage, retrieval attempts sometimes failed due to timeout errors. In these cases, simply repeating the retrieval process was often sufficient to access the data successfully. Batch processing mitigated this issue through retries, to prevent disruption to clinical services due to increased server workload, we scheduled batch jobs to run after hours when PACS traffic was much reduced. The entire image dataset retrieval was successfully completed within 3 days.

Including REB approval, data warehouse report generation and image data retrieval from PACS, all data collection was completed at our local institution in under 4 months. This approach will serve as a successful working model for future large data collection projects.

While several publicly available imaging datasets exist—such as the Stanford dataset (20) and other multimodal repositories (21)—none include V/Q scintigraphy. A recent publication by Slomka et al. provides a valuable example by detailing the development of the REFINE SPECT registry for myocardial perfusion imaging and associated clinical data (22). However, even in such efforts, data collection typically involves a labor-intensive manual workflow: patient identification, DICOM extraction from PACS, anonymization of DICOM files, de-identification of clinical reports, annotation, and database construction. This manual approach not only limits scalability but also hampers timely development of AI solutions. In contrast, VQ4PEDB introduces an automated and standardized pipeline that streamlines these processes. For instance, while PACS retrieval remains largely manual across institutions, VQ4PEDB fully automated the image data retrieval and deidentification tasks, significantly reducing human overhead. Moreover, many existing databases are static and closed to external contributions, impeding collaborative expansion and real-world adaptability. This automated pipeline also substantially reduces human exposure to patients' PHI and PII in clinical report text, thereby enhancing data privacy and security during the curation process. Our infrastructure is designed to be extensible, collaborative, and compatible with modern machine learning workflows—facilitating direct ingestion into deep learning pipelines. Thus, to our knowledge, VQ4PEDB is not only a first-of-its-kind multimodal V/Q dataset, but also a blueprint for constructing automated, scalable, and collaborative imaging registries tailored for AI-driven research.

### Annotations

PIOPED scores were already present in few clinical reports, however, PIOPED is not commonly reported for chronic patients. Therefore, annotators were tasked to reevaluate and assign a new PIOPED probability for every study, including chronic cases. The modified PIOPED criteria does not have normal perfusion with ventilation reverse mismatches category. Therefore, we included a category to capture the scenario when there is an absence of evidence for PE (i.e., normal probability without segmental perfusion defects) but defects are evident on the ventilation portion. In endpoints such as image registration, broncho pulmonary atlas mapping, and count enhancements, this category could be used to exclude cases with defects on ventilation studies.

Nuclear medicine physicians commonly report PIOPED scores which are neither quantitative nor need accurate segmentation of defects. In this study, annotators were asked to annotate segmental perfusion defects and ignore nonsegmental perfusion defects. Nonsegmental perfusion defects are consequent of other process, irregularly shaped, and do not correlate with bronchopulmonary anatomic segments. They are generally not wedge-shaped and may or may not be pleural-based (23). Partially mismatched defects were also expected to be annotated, which were another source of discrepancy among annotators, which is commonly exacerbated by blurring of images, particularly on ventilation scans. Although physicians were tasked with annotating vascular defects of any size (segmental and subsegmental), the most controversial cases were those with small lesions at the periphery of the lungs or cardiac silhouette and can be related to artifacts or cardiac cavity. This is a limitation, as annotators have restricted access to ancillary data such as, CXR which may affect the accuracy of their assessments. Inconsistent labelling of such lesions in the training dataset can confuse the AI models. The two first training datasets helped annotators to learn the morphological characteristics for appropriate annotation. As illustrated in Figure 7, most abnormal cases in the database exhibit only a few (1–3) defects across the six projections, reflecting an underrepresentation of defects in the dataset. This imbalance may pose a limitation for AI models, as the segmentation task requires a sufficient number of representative cases to ensure robust training and generalization. Employing a combination of data augmentation and diversity-enhancement strategies could be used to improve the representational diversity of the dataset. These include classical augmentations (e.g., spatial/geometric transformations), generative augmentation using Generative Adversarial Networks (GAN) (24), and manually inserting pathologies which is time-intensive (25). Another approach could use weighting of the loss function or a selection of image subsets. Nevertheless, the data from TOH and UHT is comprised of consecutive patients in the clinic and thus represents the true distribution of cases at these clinics.

Consequently, the development of a large and diverse database is essential to enable effective automatic segmentation of segmental perfusion defects and reliable detection of PE. While invasive angiography, the gold standard for diagnosing PE, is not being performed anymore, V/Q scintigraphy sensitivity was validated against angiography within PIOPED study (8, 26). There is a wide range of inter- and intra-observer variability in reading V/Q images which makes it potential candidate to benefit from automated and reproducible modern AI algorithms.

One of the key technical improvements to the annotation setup on the V7 platform was the provision of clinical reports for annotators, which was implemented for patients annotated after the initial bulk dataset, including referral physician's final impression, location of potential V/Q mismatch site, comments on heterogeneity/ homogeneity of V/Q images. Although annotators were instructed to record their independent judgment, the availability of these reports effectively served as a form of second-reader consultation.

The V7 Darwin platform is a web-based AI annotation tool designed primarily for natural image and video data, and it has been adapted for certain medical imaging workflows. While it supports a variety of file formats, including JPEG, PNG, TIFF, BMP, MP4, and AVI, DICOM files are not natively supported. They can, however, be ingested after conversion to standard 2D image formats. Annotations can be exported in multiple industry-standard formats such as JSON (Darwin native), COCO, Pascal VOC, and YOLO, enabling interoperability with machine learning frameworks like PyTorch and TensorFlow. Data upload can be performed via the web interface, CLI, or through API endpoints, and the platform supports integration into custom pipelines through its RESTful API. Once uploaded, datasets are organized into version-controlled projects that allow for structured annotation workflows and collaboration. Although V7 Darwin includes basic image manipulation features, its windowing capabilities are limited, particularly for grayscale medical imaging; annotators must adjust brightness and contrast on a per-image basis, and global or modality-specific presets are not supported. The platform includes a built-in quality control system that allows project leads to reject annotations and provide targeted feedback at the image or projection level, which is particularly useful for medical datasets. Real-time collaboration, version tracking, and audit logs are supported, alongside role-based access control. V7 Darwin is hosted on secure AWS infrastructure with encryption at rest and in transit, and while it is GDPR-compliant, HIPAA compliance is available through enterprise agreements with appropriate data handling terms.

### Data ingestion

QA revealed our unstructured text data to be properly deidentified, highlighting the effectiveness of our multiple layers of de-identification approaches. Structured DICOM data from hospital sources proved straightforward to robustly de-identify using our strategy. Also, during our QA process, various nonstructured DICOM tags, such as series description, used during splitting process were identified and addressed accordingly to preserve integrity of workflow.

Deep Lake database stands out as an indispensable tool for our study due to its specialization in handling diverse types of raw data crucial for deep learning applications, such as images, text, and other unstructured formats. This specialized database transforms raw data into a deep learning native sensorial storage format, optimizing accessibility and efficiency for model training across networks. Loading data using queries is efficient in terms of both time and computational resources. Moreover, Deep lake's integration capabilities enable advanced functionalities such as training and fine-tuning using state-of-the-art data loading mechanisms tailored for AI frameworks. This allows seamless connectivity and enhances the ability to harness diverse data types effectively in deep learning studies, where data loading and preprocessing won't be a bottleneck of an experiment.

Building such a comprehensive database inevitably comes with significant drawbacks, including substantial time and effort, financial costs, complexity, and data transferring issues. The development process involves extensive customization to align functionalities with the expected data structure. The complexity of managing a comprehensive database can pose challenges in data integration, system performance, and overall management.

Additionally, ensuring robust data security to protect against breaches and unauthorized access adds another layer of difficulty and expense. As the database grows, scalability issues may arise, requiring careful planning to accommodate increased data volume and user load. To address security concerns, we have stored our data in secure Microsoft Azure storage containers. For systems with low-speed internet connections, data loading to models could become a bottleneck. In such cases, an alternative approach is to use a local database with provisions to synchronize it with an online database, allowing for efficient data access and collaboration. Considering both the benefits and drawbacks, we recommend Deep Lake for only large-scale, long-term, and multi-institutional projects.

Microsoft Azure encrypts data both at rest and in transit. The administrator of the Azure storage account can restrict access based on geographic location and user identity. Access to storage accounts is granted through shared access signatures (SAS) with defined expiry dates and permission levels tailored to specific roles (e.g., data providers, students, annotators). Any data processed locally is protected by the secure network infrastructure at the core lab (TOH).

All patient data used in this study were fully de-identified prior to annotation, ensuring that neither patients could be re-identified. Furthermore, in the finalized database annotators are anonymous with no way to link their real or study identities to specific annotations. Annotator participation was entirely voluntary, and annotators were informed they could withdraw at any time, but their completed annotations may be retained. Their engagement in the task was considered implied consent and they were financial reimbursed for completed annotations.

The VQ4PEDB dataset is distinguished by its depth and diversity, encompassing data from multiple centers. However, given that the majority of cases originate from TOH, it is essential to critically assess AI models trained on this dataset for potential biases stemming from TOH-specific acquisition protocols. Thus our ingestion pipeline was designed to accommodate future data from other sources.

### Missing data

TOH acquires standard six projections, while UHT and Segmed Inc. data include eight projections. Unlike other centers TOH database includes several ancillary data such as nuclear medicine physician narratives and impressions, clinical reports and imaging files from CTPA, pre-scan ultrasound impressions for DVT, D-dimer lab test results, and thrombosis unit reports. These additional data provide a comprehensive resource for various applications, including multimodal diagnostic studies, validation of AI models for cross-modality integration, and research into clinical pathways for thrombosis management. We excluded studies that were missing either the ventilation or perfusion component in planar or SPECT scans, as well as those with fewer than six planar projections. Annotation of SPECT V/Q images has not yet been completed but will be integrated into the dataset upon finalization, further enhancing its utility for research and clinical applications.

### Limitations

One potential limitation of VQ4PEDB is the absence of chest x-rays (CXRs), which are commonly used in clinical practice to help assess PE probability through the PIOPED criteria. Since PIOPED classification depends on identifying triple matches or mismatches (including CXR findings) the absence of CXR data could influence the interpretive accuracy of certain cases. However, the primary objective of our annotation process was to identify V/Q mismatched vascular defects consistence with the appearance of PE, not to assign PIOPED probability scores. Therefore, VQ4PEDB could be used to design an AI workflow that specifically could detect these mismatched defects using only V/Q images, thereby enabling a segmentation pipeline that operates independently of ancillary imaging. Nonetheless, the lack of CXR input may have occasionally contributed to overcalling of defects by annotators, as CXRs are typically used to rule out alternative diagnoses that can mimic perfusion defects. Importantly, this remains an addressable limitation: our fully automated and scripted workflow is designed to be readily extensible. Should we choose to incorporate CXR data, retrieval and integration into the Deep Lake database—*via* accession number alignment and the pipeline shown in Figure 3—would require minimal additional effort.

An important challenge we faced during the creation of the database was the difficulty in adjusting windowing parameters (brightness and contrast) while annotating images in the Darwin platform. Annotators were unable to apply consistent windowing settings across all projections of a patient nor between studies nor customize the default image visualization configuration, which hindered optimal visualization during the annotation process. Proper windowing is essential for accurate interpretation of V/Q scans, and the inability to dynamically and intuitively adjust these settings—compared to standard nuclear medicine viewing software—posed a notable challenge. Although some modifications were implemented between annotation phases to improve this functionality, the solution remained somewhat cumbersome and lacked the fluidity and responsiveness of dedicated imaging platforms. While we do not believe this limitation compromised the quality of the annotations, it may have significantly impacted the efficiency of the annotation process and contributed to annotator fatigue. V7 Darwin was not originally designed with medical imaging as its primary focus, which contributes to its limited windowing capabilities. In future work, we aim to address this limitation through close collaboration with the platform's technical team during interim phases of the annotation process.

## Conclusion

This study established a multisource database, the VQ4PEDB, annotated the V/Q scintigraphic images, and documented the challenges and solutions encountered during its development. The VQ4PEDB database represents a significant resource for advancing PE diagnosis and management. It holds immense potential advancing multimodal diagnostic research, assessing AI models for cross-modality applications, and facilitating comprehensive investigations into clinical workflows for thrombosis management. This work not only highlights the importance of curated datasets in medical imaging but also provides a roadmap for addressing challenges in large-scale data annotation and integration. Availability of this dataset will foster the development of AI-driven lung V/Q applications.

## Data Availability

The original contributions presented in the study are included in the article/Supplementary Material, further inquiries can be directed to the corresponding author.
