# Correction: On the construction of a large-scale database of AI-assisted annotating lung ventilation-perfusion scintigraphy for pulmonary embolism (VQ4PEDB)

**DOI:** 10.3389/fnume.2025.1671281

**Published:** 2025-08-12

**Authors:** Amir Jabbarpour, Eric Moulton, Sanaz Kaviani, Siraj Ghassel, Wanzhen Zeng, Ramin Akbarian, Anne Couture, Aubert Roy, Richard Liu, Yousif A. Lucinian, Nuha Hejji, Sukainah AlSulaiman, Farnaz Shirazi, Eugene Leung, Sierra Bonsall, Samir Arfin, Bruce G. Gray, Ran Klein

**Affiliations:** ^1^ Department of Physics, Carleton University, Ottawa, ON, Canada; ^2^ Electrical Engineering and Computer Science, University of Ottawa, Ottawa, ON, Canada; ^3^ Research & Development, Jubilant DraxImage, Kirkland, QC, Canada; ^4^ Division of Nuclear Medicine and Molecular Imaging, Faculty of Medicine, University of Ottawa, Ottawa, ON, Canada; ^5^ Department of Nuclear Medicine and Molecular Imaging, The Ottawa Hospital, Ottawa, ON, Canada; ^6^ Department of Radiology and Nuclear Medicine, Hôpital Maisonneuve-Rosemont, Montréal, QC, Canada; ^7^ McGill University Faculty of Medicine, Rue de la Montagne, Montréal, QC, Canada; ^8^ Department of Nuclear Medicine, Jewish General Hospital, Montreal, QC, Canada; ^9^ Department of Medical Imaging, St. Michael’s Hospital, Unity Health Toronto, Toronto, ON, Canada

**Keywords:** database, image annotation, crowdsourcing, ventilation-perfusion scintigraphy, pulmonary embolism

We have incorrectly opined about the performance of a private company's third party software. The performance related to Canadian zip codes as well as other types of data was due to a user error and improper configuration of the tool by the authors. We have made the following edits:

A correction has been made to the section “Methods, Anonymization of DICOM files and de-identification of clinical reports, second paragraph”. The correct paragraph reads:

“For clinical report texts, we adopted and compounded the effect of the following three independent approaches as a conservative de-identification strategy: (1) Segmed Inc.'s Python-based web server was used to remove PII/PHI from clinical reports, (2) RegEx rules were used to remove Canadian formatted addresses and postal codes in Python, and (3) resulting texts were fed to a Microsoft Copilot agent that was instructed to list suspected people names, addresses, street names, 5–8 digit numbers, business names, clinic names and occupations. The agent was further prompted to ignore medical terms. The resulting terms were then manually screened for relevance, the terms were searched for in the text and then replaced with “[Anon]”.”

A correction has been made to the section “Discussion, Data Ingestion, first paragraph”. The correct paragraph reads:

“QA revealed our unstructured text data to be properly de-identified, highlighting the effectiveness of our multiple layers of de-identification approaches. Structured DICOM data from hospital sources proved straightforward to robustly de-identify using our strategy. Also, during our QA process, various non-structured DICOM tags, such as series description, used during splitting process were identified and addressed accordingly to preserve integrity of workflow.”

Original reference 27 has been removed.

The original version of this article has been updated.

