# Supplementary material for: On the construction of a large-scale database of AI-assisted annotating lung ventilation-perfusion scintigraphy for pulmonary embolism (VQ4PEDB)
Source: Front Nucl Med. 2025 Jul 17;5:1632112. doi: 10.3389/fnume.2025.1632112 (PMC12310601; doi:10.3389/fnume.2025.1632112)
Supplement: Supplementary file 1 [file Table1.docx]

| **Table 1: V/Q scans** | |
| --- | --- |
| **Variable or Field Name** | **Definition** *(Numerator and Denominator if applicable)* |
| MRN | Patient MRN |
| PatientID | De-identified Epic patient ID |
| PatientAge | Patient age at V/Q exam |
| Sex | Patient sex |
| GenderIdentity | Patient gender identity, if reported |
| OrderingDept | Department that ordered the V/Q scan.  NOTE: For orders placed before Epic go-live (but that occurred on or after 01June2019). |
| OrderDtm | Datetime the V/Q exam order was placed |
| ProcedureDtm | Datetime of the V/Q scan |
| AccessionNumber | Accession number for the V/Q scan |
| ExamReason | Reason for the V/Q exam |
| Narrative | Narrative section of the text report (minus the findings section) |
| Findings | Findings section of the text report.  Only populated if the narrative section had the keyword "FINDINGS:". |
| Impression | The impression section of the text report |

| **Table 2: CPTA scans** | |
| --- | --- |
| **Variable or Field Name** | **Definition** *(Numerator and Denominator if applicable)* |
| MRN | Patient MRN |
| PatientID | De-identified Epic patient ID |
| ProcedureName | Name of the procedure performed |
| ProcedureDtm | Datetime of the scan |
| AccessionNumber | Accession number for the scan NOTE: accession number is unavailable for for pre-Epic scans |
| Report | The entire text report (narrative, findings, impression) NOTE: for pre-Epic text reports, it was not feasible to separate them into narrative vs findings vs impression due to inconsistent labelling of each section, so instead the entire report is provided |
| Narrative | Narrative section of the text report (for Epic scans only) |
| Impression | The impression section of the text report (for Epic scans only) |

| **Table 3: Ultrasound for leg DVT scans** | |
| --- | --- |
| **Variable or Field Name** | **Definition** *(Numerator and Denominator if applicable)* |
| MRN | Patient MRN |
| PatientID | De-identified Epic patient ID |
| ProcedureName | Name of the procedure performed |
| ProcedureDtm | Datetime of the scan |
| Impression | The impression section of the text report (for Epic scans only) |

| **Table 4: D-dimer lab tests** | |
| --- | --- |
| **Variable or Field Name** | **Definition** *(Numerator and Denominator if applicable)* |
| MRN | Patient MRN |
| PatientID | De-identified Epic patient ID |
| SpecimenCollectionDtm | Datetime when the specimen was collected |
| LabResultDtm | Datetime of the lab result |
| Result | Lab result |
| Units | Units of measure for the lab result |

| **Table 5: Thrombosis follow-up notes** | |
| --- | --- |
| **Variable or Field Name** | **Definition** *(Numerator and Denominator if applicable)* |
| MRN | Patient MRN |
| PatientID | De-identified Epic patient ID |
| ThrombosisVisitDate | Date of the thrombosis visit |
| ThrombosisNote | Note from thrombosis provider |
