# Supplementary material for: On the construction of a large-scale database of AI-assisted annotating lung ventilation-perfusion scintigraphy for pulmonary embolism (VQ4PEDB)
Source: Front Nucl Med. 2025 Jul 17;5:1632112. doi: 10.3389/fnume.2025.1632112 (PMC12310601; doi:10.3389/fnume.2025.1632112)
Supplement: Supplementary file 2 [file Table2.docx]

list_tags=['InstitutionName',

'InstitutionAddress',

'ReferringPhysicianName',

'ReferringPhysicianAddress',

'ReferringPhysicianTelephoneNumbers',

'ReferringPhysicianIdentificationSequence',

'ConsultingsPhysicianName',

'ConsultingPhysicianIdentificationSequence',

'PrivateDataElement',

'PhysiciansOfRecord',

'PhysiciansOfRecordIdentificationSequence',

'PerformingPhysicianName',

'PerformingPhysicianIdentificationSequence',

'NameOfPhysiciansReadingStudy',

'PhysiciansReadingStudyIdentificationSequence',

'OperatorsName',

'OperatorIdentificationSequence',

'PatientName',

'PatientID',

'IssuerOfPatientID',

'PatientBirthTime',

'PatientBirthDateInAlternativeCalendar',

'PatientDeathDateInAlternativeCalendar',

'PatientDeathDateInAlternativeCalendar',

'PatientPrimaryLanguageCodeSequence',

'OtherPatientIDs',

'OtherPatientNames',

'OtherPatientIDsSequence',

'PatientBirthName',

'PatientAddress',

'InsurancePlanIdentification',

'PatientMotherBirthName',

'MedicalRecordLocator',

'CountryOfResidence',

'RegionOfResidence',

'PatientTelephoneNumbers',

'PatientTelecomInformation',

'EthnicGroup',

'PatientReligiousPreference',

'PatientSpeciesDescription',

'ResponsiblePerson',

'ResponsibleOrganization',

'PatientComments',

'ConsultingPhysicianIdentificationSequence',

'PerformingPhysiciansName',

'OperatorsName',

'OperatorIdentificationSequence',

'OperatorAddress',

'StationName',

'InstitutionName',

'InstitutionAddress',

'InstitutionCodeSequence',

'PatientLastMenstrualDate',

'MedicalAlerts',

'PerformingPhysicianIdentificationSequence'

'TechnologistOrganizationName',

'TechnologistAddress',

'TechnologistsTelephoneNumber',

'InstitutionCodeSequence',

'InstitutionalDepartmentName',

'PerformingOrganizationName',

'RequestingService',

'MilitaryRank',

'ReferringPhysicianIdentificationSequence',

'PhysicianOfRecordRelationship',

'TechnologistResidence',

'StationName',

'PersonNameCodeSequence',

'AdmittingDiagnosesDescription',

'AdmissionID',

'ReferringPhysicianName',

]

dcm.remove_private_tags()
